# Salmonellosis outbreak associated with the consumption of food at a wedding in an urban restaurant in Kazakhstan: a retrospective cohort study

**DOI:** 10.1186/s12879-024-10382-4

**Published:** 2024-12-25

**Authors:** Saya Gazezova, Dilyara Nabirova, Michelle Waltenburg, Maral Rakhimzhanova, Manar Smagul, Lena Kasabekova, Roberta Horth

**Affiliations:** 1 Central Asia Field Epidemiology Training Program, Asfendiyarov Kazakh National Medical University, Almaty, Kazakhstan; 2 Scientific and Practical Center for Sanitary and Epidemiological Expertise and Monitoring, Almaty, Kazakhstan; 3 Division of Global Health Protection in Central Asia, United States Centers for Disease Control and Prevention, Almaty, Kazakhstan; 4 Division of High-Consequence Pathogens and Pathology, United States Centers for Disease Control and Prevention, Atlanta, USA; 5 Ministry of Health of Kazakhstan, Astana, Kazakhstan; 6 National Center for Public Health, Astana, Kazakhstan

**Keywords:** Foodborne outbreak, Outbreak investigation, *S. Enteritidis*, Kazakhstan

## Abstract

**Background:**

From June 13–16, 2022, a regional epidemiological department in Kazakhstan reported an increase in acute gastroenteritis cases among people who consumed food from a wedding at a restaurant. An investigation was initiated to determine factors associated with acute intestinal infection and prevent further illness.

**Methods:**

The investigation team conducted a retrospective cohort study among people who consumed event food. Participants were classified as a case if they were acutely ill with diarrhea, vomiting, fever, vomiting, or weakness from June 13–18. We interviewed people to collect information on demographics, symptoms, and food exposures at the event. We calculated food-specific attack rates and estimated adjusted relative risks (aRR) using multivariable Poisson regression, which was adjusted for sex, age, and foods consumed. Patient stool and gastric lavage samples, leftover food, and restaurant environmental samples were collected for bacterial culture and chemical analysis.

**Results:**

Of the 138 participants, 66 became ill; the attack rate was 48%. The most reported symptoms were diarrhea (92%), abdominal pain (91%), and fever (89%). Symptom onset occurred between 6 h and 4 days after the event (median = 1 day). Overall, 50 (76%) cases were hospitalized; no deaths were reported. In bivariable analysis, a greater proportion of cases than non-cases ate honey cake (89% vs. 13%, *p* < 0.01), and 45% of cases ate leftovers compared with 11% of non-cases (*p* < 0.01). In multivariable analysis, honey cake was the only risk factor associated with illness (aRR = 7.8, 95% confidence interval = 3.5–20.1, *p* < 0.01). Honey cakes, which use raw eggs in cream layers, had been stored at room temperature for three days before the event. *Salmonella enterica* serovar Enteriditis (S. Enteritidis) was isolated from all patient stool samples (49/49, 100%) and honey cake samples (2/2, 100%). *Staphylococcus aureus* was detected in 92% (35/38) of patient gastric lavage samples.

**Conclusion:**

*S. Enteritidis* was this outbreak’s most probable etiological agent based on clinical manifestations and isolation from participant and honey cake samples. The improper storage of cakes containing raw eggs was a key contributing factor. Leftover event food was discarded, and the restaurant was closed for disinfection. Future outbreaks could be prevented by increased food safety awareness.

**Supplementary Information:**

The online version contains supplementary material available at 10.1186/s12879-024-10382-4.

## Introduction

Nontyphoidal Salmonella causes approximately 150 million illnesses and 60,000 deaths globally each year [1]. *Salmonella* is one of the leading bacterial causes of diarrhea and remains a pressing public health problem in many developed and developing countries. Transmission typically occurs from eating contaminated foods and can occur from drinking contaminated water or contact with people with diarrheal illness. *Salmonella enterica* serovar Enteritidis (S. Enteritidis) is the most frequent causative agent of nontyphoidal human salmonellosis in many countries, including Kazakhstan, and is commonly associated with foodborne transmission [2–4].

In 2022, Kazakhstan recorded 23 outbreaks of salmonellosis, representing a twelvefold increase compared with 2021, which had only 2 outbreaks, but below the number recorded in 2019, which had 26 outbreaks [5]. Foodborne outbreak investigations in Kazakhstan fall under the jurisdiction of the local departments of sanitary and epidemiological control. When these outbreaks are large, local departments receive additional support from the National Scientific and Practical Center for Sanitary and Epidemiological Expertise and Monitoring.

From June 13 to 16, 2022, an oblast Department of Sanitary and Epidemiological Control recorded an increase in the number of cases of acute intestinal infections. Initial epidemiological investigations determined that cases occurred among participants of a wedding held in the same restaurant. A team from the Scientific and Practical Center for Sanitary and Epidemiological Expertise and Monitoring and from the Field Epidemiology Training Program in Central Asia was deployed in the city to conduct an outbreak investigation to describe epidemiological and clinical characteristics, determine the risk factors associated with acute intestinal infection, and identify the source to control the outbreak.

## Methods

### Study design

We conducted a retrospective cohort study from June 17 to 26, 2022. The cohort was defined as all persons who consumed food served at a restaurant wedding event on June 12, 2022, including those who consumed leftover food off-site. Cases were classified as anyone who consumed food served at the event, including leftovers, who were acutely ill, hospitalized, or outpatient treatment in the period after the wedding and June 18, 2022, with signs and symptoms of diarrhea, fever (≥ 37.5 °C), vomiting, or weakness.

The list of cases was obtained from the Department of Sanitary and Epidemiological Control. The team interviewed all the event participants and those who consumed event food by reviewing the list of wedding guests provided by the organizers. A list was also prepared based on interviews with attendees of people who consumed wedding food but were not in attendance. All the interviewed attendees had consumed food. One person who prepared food for the event but did not attend the event or consume food from the event was also interviewed.

### Questionnaire tool

The team used a structured questionnaire adapted from several sources to assess demographic, epidemiological, clinical, and laboratory characteristics, which was pilot-tested before administration (Supplement 1) [6, 7]. The interviews were administered by trained epidemiologists in person or over the phone. Investigators developed a list of potential risks or exposures based on the menu from the event. Clinical and laboratory data were obtained from medical records.

### Data analysis

Participant data were collected using KoboToolbox (Cambridge, USA). The data were cleaned and analyzed using R v4.2 (Vienna, Austria). Food-specific attack rates (ARs) were calculated. Bivariable chi-square tests and multivariable Poisson regression were performed to detect associations between food exposures and acute intestinal infection using relative risks (RR) and adjusted relative risk (aRR) and 95% confidence intervals (95% CIs). Variables statistically significant in the bivariable analysis, such as age and sex, were included in the multivariable model. Statistically significant associations (*p* < 0.05) were included in the multivariable analysis.

### Laboratory testing

The results from bacterial cultures of patient fecal and gastric lavage samples were obtained from medical records for patients hospitalized at the city hospital. Nasal and throat swabs from healthy restaurant personnel and the person who prepared food for the event were also collected and tested.

### Environmental assessment

An environmental assessment and restaurant site visit was conducted by the Department of Sanitary and Epidemiological Control 2 days after the event. Samples from leftover raw ingredient materials and prepared dishes served at the event, from restaurant surfaces (e.g., plates, tables), from disinfectants used, and from tap water used at the restaurant were collected and submitted to the branch of the National Center of Expertise for bacterial culture and chemical contaminants (residual nitrate content, iodine content, measurement of antibiotic and hormone residues).

### Ethical considerations

This activity was reviewed by the CDC, deemed not research, and was conducted consistent with applicable federal law and CDC policy^1^. All the participants provided written informed consent. Parents or legal guardians gave consent to provide information about their children under the age of 18.

## Results

### Participant characteristics

We identified 182 people who attended the event or consumed food from the event, of whom 44 (24%) were excluded from the study due to refusals (*n* = 9) or absence from their residence at the time of the survey (*n* = 35). Among the remaining 138 study participants, 66 were classified as cases. The attack rate was 48% (66/138). The median age was 34 years (interquartile range: 23 years). Most participants were male (*n* = 90, 65%). Sex did not differ between case and non-case participants (*p* = 0.15) (Table 1). Cases were significantly younger than non-cases (*p* < 0.01). Cases were more likely to have consumed leftovers than non-cases (69% vs. 26%).

**Table 1:** Characteristics of people who participated in the event or consumed event food, Kazakhstan, June 2022

Characteristics	Case *n* = 66 (%)	Non-case *N* = 72 (%)	*p*-value*
Sex			
Male	39 (59)	51 (71)	ref
Female	27 (41)	21 (29)	0.15
Age group (years)			
≤ 19	28 (43)	10 (14)	ref
20–39	20 (30)	24 (33)	0.10
40+	18 (27)	38 (53)	< 0.01
Food exposure			
Only at the wedding	28 (30)	53 (74)	ref
Only leftover food	20 (42)	8 (11)	< 0.01
Both	18 (27)	11 (15)	0.02
Symptoms			NA
Diarrhea	61 (92)	0	
Abdominal pain	60 (91)	0	
Fever ≥ 37.5˚C	59 (89)	0	
Headache	58 (88)	0	
Nausea	55 (83)	0	
Chills	47 (71)	0	
Vomiting	47 (71)	0	

*P-value from maximum likelihood test from Poisson regression estimators

Overall, 76% (50/66) of cases were hospitalized. No deaths were recorded. The most commonly reported signs and symptoms were diarrhea (92%), abdominal pain (91%), fever ≥ 37.5 °C (89%), headache (88%), and nausea (83%).

**Fig. 1:**
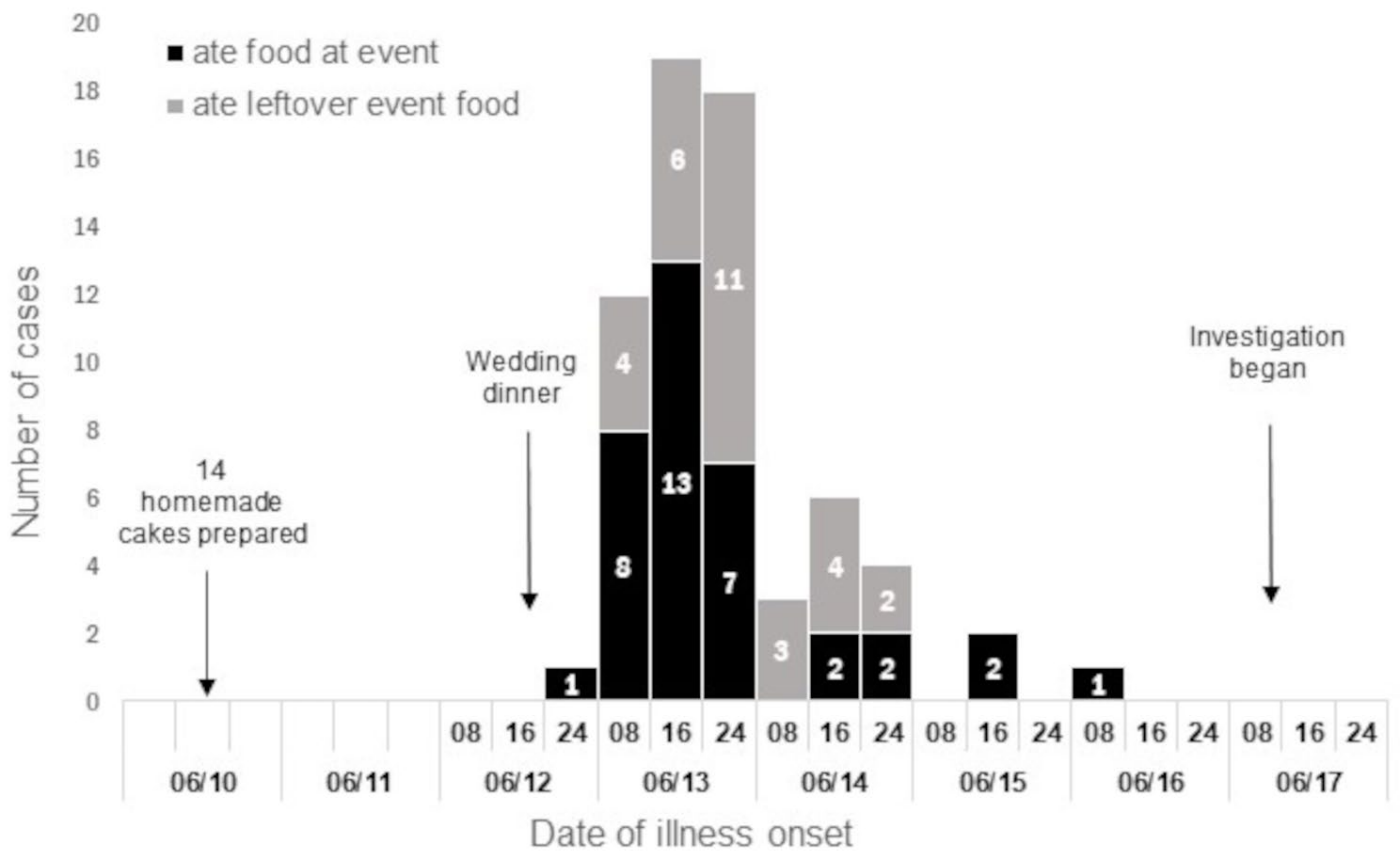
People with acute intestinal infection after a wedding event, by date and time of illness onset, Kazakhstan, June 2022 (*N* = 66)

Among the cases (*n* = 66), symptom onset occurred from June 13 to June 16, 2022, with a median time of 11 h, ranging between six hours and four days after the event (Fig. 1). Illness onset peaked on June 13, 2022, approximately 24 h after the event. The median duration of illness was 2 days (range: 1–4 days).

### Factors associated with illness

In bivariable analysis, a greater proportion of the cases than non-cases ate honey cake (89% vs. 13%, respectively, *p* < 0.01) (Table 2). Nearly half of the cases (45%) ate leftovers compared to 11% of non-cases (*p* < 0.01).

**Table 2:** Factors independently associated with acute intestinal infection after a wedding event, Kazakhstan, June 2022

Factor	Ate the food item			Did not			RR (95%CI)	Chi-square *p*-value
	Total	Cases	AR	Total	Cases	AR		
Honey cake^1^	68	59	87%	70	7	10%	8.7 (4.3–20.9)	< 0.01
Ate leftovers	52	42	81%	86	24	28%	2.9 (2.0–4.2)	< 0.01
Beshbarmak^2^	89	32	36%	49	34	69%	0.5 (0.4–0.7)	0.01
Samsa^3^	59	17	29%	79	49	62%	0.5 (0.3–0.7)	0.01
Chicken roll	40	12	30%	98	54	55%	0.5 (0.3–0.7)	0.05
Liver cake	48	22	46%	90	44	49%	0.9 (0.7–1.4)	0.80
Salad w/ beef & soy sauce	54	22	41%	84	44	52%	0.8 (0.5–1.2)	0.34
Salad w/ mayonnaise, chicken, & egg	63	25	40%	75	41	55%	0.7 (0.5–1.1)	0.21
Chicken kebab	47	17	36%	91	49	54%	0.7 (0.4–1.0)	0.16

AR: attack rate; RR: relative risk; CI: confidence interval

1 A layered dessert sweetened with honey and filled with a cream that often includes raw eggs

2 A traditional Kazakh dish made of boiled meat served over flat noodles and topped with onion sauce

3 A traditional ground meat dumpling

In multivariable analyses that adjusted for sex, age, and other foods independently associated with illness, the only exposure significantly associated with acute intestinal infection was consumption of honey cake (aRR = 8.0, 95% CI = 3.6–20.7, *p* < 0.01) (Table 3). In a sub-analysis that included only people who had attended the wedding event, consumption of honey cake continued to be the only risk factor associated with illness (aRR = 9.2, 95% CI = 3.8–26.0, *p* < 0.01).

**Table 3:** Multivariable analysis of factors associated with acute intestinal infection after a wedding event, Kazakhstan, June 2022

Group		Entire cohort (*n* = 138)				Wedding attendees (*n* = 100)		
Risk factors		aRR	95%CI	*p*-value		aRR	95% CI	*p*-value
Female sex		0.9	0.6–1.6	0.82		0.8	0.4–1.7	0.58
Age group (years)								
≤ 19		Ref				Ref		
20–39		0.9	0.5–1.7	0.71		1.0	0.4–2.6	0.94
40+		0.8	0.4–1.6	0.52		0.8	0.3–2.1	0.62
Exposure type								
Only wedding food		Ref				Ref		
Only leftover food		1.0	0.4–3.7	0.97		-	-	-
Both wedding and leftover food		1.2	0.6–2.5	0.64		1.1	0.5–2.4	0.73
Honey Cake^1^								
Ate leftovers		8.0	3.6–20.7	0		9.2	3.8–26	< 0.01
Beshbarmak^2^		1.2	0.5–4.3	0.69		1.3	0.5–4.5	0.68
Chicken roll		0.7	0.3–1.4	0.28		0.7	0.3–1.3	0.26
Samsa^3^		0.9	0.4–1.8	0.75		0.9	0.4–1.8	0.74

CI: confidence interval; aRR: adjusted risk ratio, *p*-value: Maximum likelihood test of Poisson regression estimators

1 A layered dessert sweetened with honey and filled with a cream that often includes raw eggs

2 A traditional Kazakh dish made of boiled meat served over flat noodles and topped with onion sauce

3 A traditional ground meat dumpling

### Laboratory testing

Fecal or gastric lavage test results were available for 76% (50/66) of cases. Of fecal samples from 49 cases, *S.* Enteritidis was confirmed in 49 (100%) samples. Of gastric lavage samples collected from 38 cases, *Staphylococcus aureus* was identified in 35 (92%) samples. Altogether of the 50 cases’ stool or gastric lavage samples, both *S.* Enteritidis and *Staphylococcus aureus* were detected in 34 (68%), only *S.* Enteritidis were detected in 15 (30%), and only were detected *Staphylococcus aureus* in 1 (2%).

Fourteen samples were collected from leftover ingredients and prepared dishes (Supplement 2). The following pathogens were detected: *E. coli* in cooked liver pancake, *E. coli* and *Enterobacter spp.* in uncooked mushrooms, *E. coli*,* Enterobacter spp*, and *S.* Enteritidis in two samples of honey cake, and *E. coli* in uncooked horsemeat which is an ingredient of beshbarmak (a traditional Kazakh noodle, onion, and boiled meat dish). No trace nitrites or iodine were detected in these water and food samples. Among 100 samples collected from restaurant food preparation and serving surfaces, *E. coli* was detected in 24 (24%) samples, including on plates, tables, and dish racks. No pathogens were detected in nasal and throat swabs collected from the eight restaurant personnel tested, and none of them had any recent illness before the wedding.

### Environmental assessment

The environmental assessment and site visit revealed that the restaurant did not have a valid sanitary and epidemiological certificate, which is required by law to operate and serve food. Furthermore, no restaurant personnel held valid health certificates that would permit them to work in a restaurant kitchen. The restaurant had been operating for approximately one month prior to the event. Inspection of the premises identified that the production line of the kitchen had technological equipment in good working order, four refrigerators at adequate temperatures (2 to 4 °C), and two freezers at unmonitored temperatures due to a lack of thermometers. No defects were noted on the surfaces of the equipment in the kitchen.

An in-depth assessment of food items independently associated with illness revealed that the 14 honey cakes, approximately 30 cm in diameter, served at the event were prepared in the home of a wedding attendee relative who had not attended the event nor consumed food from the event. The honey cakes, which were prepared using raw eggs in the cream layers, had been stored for three days prior to the event. The preparer’s private kitchen did not have adequate refrigerator space to store all the honey cakes; therefore, some honey cakes were stored at room temperature.

The honey cakes were delivered to the restaurant the morning of the event; however, the restaurant kitchen did not have adequate refrigerator space to store all the honey cakes. As a result, some cakes were stored at room temperature until they were served at approximately 9 pm. The individual who prepared the honey cakes did not develop any symptoms of acute intestinal infection. The samples were collected from this individual and were negative by microbiological testing. The individual did not hold a valid health certificate to prepare food for public consumption. All other food served at the event was prepared by restaurant cooks on-site.

### Outbreak investigation response

All leftover food from the event was discarded, and the restaurant environment was disinfected. The restaurant was closed until all health certificates could be obtained and the restaurant could be reinspected by public health officials. Recommendations were made to the restaurant to ensure that food safety measures were followed, including prohibiting the entry of food that was prepared at home or by individuals who do not hold a valid health certificate for food preparation. Restaurant personnel were also educated on compliance with proper storage requirements, including temperature conditions and the commodity proximity of products. The public was informed about food safety through public awareness campaigns on local news channels. There were no other epidemiologically linked cases in the city following these efforts. All cases fully recovered.

## Discussion

We conducted a rapid investigation of a foodborne outbreak affecting nearly half (66/138) of the attendees of a wedding event held at a restaurant in Kazakhstan in June 2022. The consumption of improperly refrigerated homemade honey cake was identified as the main risk factor associated with acute intestinal infection. Honey cake is a traditional cake that contains cream usually made with raw eggs in between cooked cake layers. This finding was strengthened by the identification of *S.* Enteritidis in samples collected from participants and leftover honey cake. While both *S.* Enteritidis and *Staphylococcus aureus* were identified in patient samples, *S.* Enteritidis is more likely the etiological agent of this outbreak based on clinical manifestations. The reported clinical manifestations (diarrhea, abdominal pain, fever) and symptom onset (between six hours and four days after the event) are more consistent with infection with S. Enteritidis compared with *Staphylococcus aureus* [1, 8]. Staphylococcal gastrointestinal illness is caused by the consumption of foods contaminated with toxins produced by *Staphylococcus aureus* and is characterized by a sudden onset of nausea, vomiting, and stomach cramps with or without diarrhea. Symptoms typically develop 30 min to eight hours after the consumption of an item contaminated by the toxin and last no longer than one day. Furthermore, *S.* Enteritidis was isolated from honey cakes, which was identified as the main risk factor associated with illness in this outbreak. However, given the gaps in food hygiene observed, we cannot rule out that *S. aureus* and other bacteria also did not contribute to the outbreak.

Multiple factors may have contributed to honey cake contamination with *Salmonella*. First, honey cakes contained raw eggs, a known risk factor for acquiring *Salmonella* infection [9, 10]. Honey cakes use thin layers of baked cake with a thin spreading of cream, made with raw eggs, in between layers. Second, honey cakes should be eaten or refrigerated promptly after preparation; cakes should be refrigerated within two hours after preparation until they are consumed. As the honey cakes implicated in this outbreak were prepared in a private home kitchen that did not have sufficient refrigeration space, some honey cakes were stored at room temperature for up to two days until they were delivered to the restaurant. The improper storage of honey cakes also occurred at the restaurant due to limited refrigeration space. Pathogenic bacteria, such as *Salmonella spp*., grow more rapidly at room temperature. Leftover foods are often left at room temperature. Bacteria would have continued to multiply in leftover foods that wedding guests took home and ate after the wedding or shared with others. In our study 7 out of 10 people who consumed leftovers were infected and developed symptoms. Leftover foods also included honeycake. This explains the higher risk among people who ate leftovers.

*Salmonella* has been implicated in large foodborne outbreaks globally [11]. These outbreaks occur in various settings, but restaurants or commercial food serving establishments are primary locations [11–13]. A substantial proportion of foodborne outbreaks have been attributed to *Salmonella*, highlighting its impact on public health in terms of the number of cases, hospitalizations, and deaths [14]. *Salmonella* outbreaks are often linked to the consumption of contaminated eggs and egg-containing foods. In the European region, egg and egg products continue to be the highest risk foods for *Salmonella* outbreaks [5]. Wedding events have been identified as common venues for foodborne outbreaks, particularly those involving raw or undercooked eggs. For instance, a large outbreak occurred at a wedding in Kyrgyzstan, where over a hundred attendees reported gastrointestinal illnesses, with raw eggs suspected as the source [15]. This incident underscores the broader issue of foodborne diseases linked to eggs, which have been a persistent problem [9]. People who prepare foods that require raw eggs could consider using alternative recipes that do not require the use of raw eggs to reduce the risk of *Salmonella* contamination. Foods made with raw eggs, when labeled, may be informative for consumers with high risk of severe illness.

Our study also found that several food and environmental samples were contaminated with other pathogenic bacteria, including *E. coli*. While it is common to detect *E coli* on restaurant surfaces [16], the extent of contamination across different surfaces and food types was unexpected and was likely the result of poor food hygiene practices. We therefore cannot rule out that other pathogenic bacteria contributed to this outbreak.

This investigation was subject to a few limitations. First, not all people who attended the event or consumed event food were included in the retrospective cohort study due to refusals or absences from residences at the time interviewers were conducted. Therefore, there might have been more cases than we identified. Second, the raw ingredients used to prepare the honey cakes had been discarded at the time the environmental assessment occurred and were unavailable for further analysis. Therefore, we cannot definitively conclude if preparation (contaminated ingredients and/or cross-contamination), inappropriate storage, or a combination of the two was the mechanism that led to honey cake contamination with *Salmonella*. This also limits the ability to conduct further tracebacks to the source of the products used for the cake. Lastly, whole-genome sequencing (WGS) was not performed of isolated bacteria. It is therefore not possible to directly link *Salmonella* detected in participant and food samples. WGS is not routinely performed in Kazakhstan, including in foodborne outbreaks, and few laboratories in the country have the capacity for WGS.

The risk of *Salmonella* contamination in foods that traditionally use raw eggs can be reduced by increased awareness, the use of alternative recipes, and the availability of raw egg substitutes, such as pasteurized eggs and egg products, which have limited availability and are only for commercial use in Kazakhstan. Additionally, behavioral-based food safety training for commercial food preparers, on best practices for handling raw ingredients, can also help reduce the risk of foodborne transmission [17]. Risk can also be reduced by increased enforcement of policies that prohibit home-prepared foods to be served in commercial establishments, and improved monitoring of compliance with appropriate regulatory certificates and proper storage requirements. Lastly, risk can be reduced by the storage of foods at cold temperatures, which can slow bacterial growth.

Timely investigation and collaboration between epidemiologic, laboratory, and environmental sectors allowed for the rapid identification of honey cake as the probable source of this outbreak. Although multiple pathogens were identified and cannot be entirely ruled out, *S.* Enteritidis was the most probable etiological agent based on clinical manifestations and isolation from participant and honey cake samples. The improper storage of cakes was a key contributing factor. Future outbreaks can be prevented through increased awareness about food safety.

## Electronic supplementary material

Below is the link to the electronic supplementary material.

Supplementary Material 1

Supplementary Material 2

## Data Availability

The datasets generated for this study are available on request to the corresponding author.

## Footnotes

1 §See e.g., 45 C.F.R. part 46, 21 C.F.R. part 56; 42 U.S.C. § 241(d); 5 U.S.C. § 552a; 44 U.S.C. § 3501 et seq.

## Abbreviations

aRR: Adjusted Relative Risks
ARs: Attack rates
CDC: Centers for Disease Control and Prevention
CIs: Confidence Intervals
RR: Relative risks
*S.* Enteritidis: Salmonella enterica serovar Enteriditis
