# Supplementary material for: Salmonellosis outbreak associated with the consumption of food at a wedding in an urban restaurant in Kazakhstan: a retrospective cohort study
Source: BMC Infect Dis. 2024 Dec 25;24:1464. doi: 10.1186/s12879-024-10382-4 (PMC11670359; doi:10.1186/s12879-024-10382-4)
Supplement: Supplementary file 1 — Supplementary Material 1 [file 12879_2024_10382_MOESM1_ESM.docx]

**Supplement 1.**

**Questionnaire tool**

**Outbreak investigation of an intestinal infection in an urban restaurant, Kazakhstan June 2022**

| Information about data collector (interviewer) | |
| --- | --- |
| Date form completed (DD/MM/YYYY) | ⬜⬜.⬜⬜.⬜⬜⬜⬜  Day Month Year |

| А. Identification number and patient information | | |
| --- | --- | --- |
| **Question** | | **Category** |
| A1 | Patient’s last name |  |
| A2 | Patient’s first name |  |
| А3 | Sex (male=1, female=2) |  |
| А4 | Age |  |
| A5 | Date of birth (DD/MM/YYYY) | ⬜⬜.⬜⬜.⬜⬜⬜⬜  Day Month Year |
| A6 | Unique ID | ⬜⬜⬜⬜⬜⬜⬜⬜⬜⬜⬜⬜ |
| A7 | Residence address |  |

| Т. Epidemiological information | | | |
| --- | --- | --- | --- |
| **Question** | | **Category** | **Answer** |
| T1 | Were you present during a celebratory event in the "El-Tay" restaurant on 12.06.2022 | **Yes=1 No=0**  *If no, go to question Т4* |  |
| **If Yes to «Т1», specify** | | |  |
| Т2 | What you ate | | |

| *Dish type* | *Dish name* | *Response options* | | | *Answer* |
| --- | --- | --- | --- | --- | --- |
| *Appetizers* | Chicken roll | **Yes=1** | **No=0** | **Don’t remember=99** |  |
|  | Chicken kabob | **Yes=1** | **No=0** | **Don’t remember=99** |  |
|  | Chicken wings | **Yes=1** | **No=0** | **Don’t remember=99** |  |
|  | Liver cake | **Yes=1** | **No=0** | **Don’t remember=99** |  |
|  | Meat samosa | **Yes=1** | **No=0** | **Don’t remember=99** |  |
| *Salads* | General (ham, smoked chicken breast, Gouda cheese, egg, cucumber, mayonnaise) | **Yes=1** | **No=0** | **Don’t remember=99** |  |
|  | Oriental-style salad (cucumber, tomato, bell pepper, beef, garlic, soy sauce, sesame seeds, oil) | **Yes=1** | **No=0** | **Don’t remember=99** |  |
|  | Hollywood (beets, feta cheese, orange, lettuce, almond chips, lolla rosso, sauce (mustard, orange juice, seasoning)) | **Yes=1** | **No=0** | **Don’t remember=99** |  |
|  | Caucasus (tomato, cucumber, bell pepper, radish, feta cheese, greens) | **Yes=1** | **No=0** | **Don’t remember=99** |  |
| *Hot dish №1* | Beshbarmak | **Yes=1** | **No=0** | **Don’t remember=99** |  |
| *Hot dish №2* | Thai-style meat | **Yes=1** | **No=0** | **Don’t remember=99** |  |
| *Fruit platter* | Orange | **Yes=1** | **No=0** | **Don’t remember=99** |  |
|  | Sweet cherry | **Yes=1** | **No=0** | **Don’t remember=99** |  |
|  | Kiwi | **Yes=1** | **No=0** | **Don’t remember=99** |  |
|  | Green apple | **Yes=1** | **No=0** | **Don’t remember=99** |  |
|  | Red apple | **Yes=1** | **No=0** | **Don’t remember=99** |  |
| *Bread basket* | Fried breat | **Yes=1** | **No=0** | **Don’t remember=99** |  |
|  | Rye bread | **Yes=1** | **No=0** | **Don’t remember=99** |  |
|  | Wheat bread | **Yes=1** | **No=0** | **Don’t remember=99** |  |
| *Desserts* | Honey cake | **Yes=1** | **No=0** | **Don’t remember=99** |  |
|  | Whoopee Pie cake | **Yes=1** | **No=0** | **Don’t remember=99** |  |
|  | «Molochnaya devochka» cake | **Yes=1** | **No=0** | **Don’t remember=99** |  |
|  | Cookies | **Yes=1** | **No=0** | **Don’t remember=99** |  |
|  | Candy | **Yes=1** | **No=0** | **Don’t remember=99** |  |
| *Beverages* | Zerno vodka | **Yes=1** | **No=0** | **Don’t remember=99** |  |
|  | Berkutti cognac | **Yes=1** | **No=0** | **Don’t remember=99** |  |
|  | Red wine | **Yes=1** | **No=0** | **Don’t remember=99** |  |
|  | White wine | **Yes=1** | **No=0** | **Don’t remember=99** |  |

| Т3 | Did you take home leftovers? | **Yes=1** | **No=0** | **Don’t remember=99** |  |
| --- | --- | --- | --- | --- | --- |
|  | If yes, please specify? *If no, go to question F1* | | | | |

| *Dish type* | *Dish name* | **Yes=1** | **No=0** | **Don’t remember=99** |  |
| --- | --- | --- | --- | --- | --- |
| *Appetizers* | Chicken roll | **Yes=1** | **No=0** | **Don’t remember=99** |  |
|  | Chicken kabob | **Yes=1** | **No=0** | **Don’t remember=99** |  |
|  | Chicken wings | **Yes=1** | **No=0** | **Don’t remember=99** |  |
|  | Liver cake | **Yes=1** | **No=0** | **Don’t remember=99** |  |
|  | Meat samosa | **Yes=1** | **No=0** | **Don’t remember=99** |  |
| *Salads* | General (ham, smoked chicken breast, Gouda cheese, egg, cucumber, mayonnaise) | **Yes=1** | **No=0** | **Don’t remember=99** |  |
|  | Oriental-style salad (cucumber, tomato, bell pepper, beef, garlic, soy sauce, sesame seeds, oil) | **Yes=1** | **No=0** | **Don’t remember=99** |  |
|  | Hollywood salad (beets, feta cheese, orange, lettuce, almond chips, lolla rosso, sauce (mustard, orange juice, seasoning) | **Yes=1** | **No=0** | **Don’t remember=99** |  |
|  | Caucasus salad (tomato, cucumber, bell pepper, radish, feta cheese, greens) | **Yes=1** | **No=0** | **Don’t remember=99** |  |
| *Hot dish №1* | Beshbarmak | **Yes=1** | **No=0** | **Don’t remember=99** |  |
| *Hot dish №2* | Thai-style meat | **Yes=1** | **No=0** | **Don’t remember=99** |  |
| *Fruit platter* | Orange | **Yes=1** | **No=0** | **Don’t remember=99** |  |
|  | Sweet cherry | **Yes=1** | **No=0** | **Don’t remember=99** |  |
|  | Kiwi | **Yes=1** | **No=0** | **Don’t remember=99** |  |
|  | Green apple | **Yes=1** | **No=0** | **Don’t remember=99** |  |
|  | Red apple | **Yes=1** | **No=0** | **Don’t remember=99** |  |
| *Bread basket* | Fried bread rolls | **Yes=1** | **No=0** | **Don’t remember=99** |  |
|  | Rye bread | **Yes=1** | **No=0** | **Don’t remember=99** |  |
|  | Wheat bread | **Yes=1** | **No=0** | **Don’t remember=99** |  |
| *Desserts* | Honey cake | **Yes=1** | **No=0** | **Don’t remember=99** |  |
|  | Whoopee Pie cake | **Yes=1** | **No=0** | **Don’t remember=99** |  |
|  | «Molochnaya devochka» cake | **Yes=1** | **No=0** | **Don’t remember=99** |  |
|  | Cookies | **Yes=1** | **No=0** | **Don’t remember=99** |  |
|  | Candy | **Yes=1** | **No=0** | **Don’t remember=99** |  |

| Т4 | If not, did you consume leftover food | **Yes=1 No=0**  *If no, go to question Т6* |  |
| --- | --- | --- | --- |
| Т5 | Date leftover food consumed | ⬜⬜.⬜⬜.⬜⬜⬜⬜  Day Month Year | |
| Т6 | Please specify | | |

| *Dish type* | *Dish name* | **Yes=1** | **No=0** | **Don’t remember=99** | ***Answer*** |
| --- | --- | --- | --- | --- | --- |
| *Appetizers* | Chicken roll | **Yes=1** | **No=0** | **Don’t remember=99** |  |
|  | Chicken kabob | **Yes=1** | **No=0** | **Don’t remember=99** |  |
|  | Chicken wings | **Yes=1** | **No=0** | **Don’t remember=99** |  |
|  | Liver cake | **Yes=1** | **No=0** | **Don’t remember=99** |  |
|  | Meat samosa | **Yes=1** | **No=0** | **Don’t remember=99** |  |
| *Salads* | General salad (ham, smoked chicken breast, Gauda cheese, egg, cucumber, mayonnaise) | **Yes=1** | **No=0** | **Don’t remember=99** |  |
|  | Oriental-style salad (cucumber, tomato, bell pepper, beef, garlic, soy sauce, sesame seeds, oil) | **Yes=1** | **No=0** | **Don’t remember=99** |  |
|  | Hollywood salad (beets, feta cheese, orange, lettuce, almond chips, lolla rosso, sauce (mustard, orange juice, seasoning) | **Yes=1** | **No=0** | **Don’t remember=99** |  |
|  | Caucasus salad (tomato, cucumber, bell pepper, radish, feta cheese, greens) | **Yes=1** | **No=0** | **Don’t remember=99** |  |
| *Hot dish №1* | Beshbarmak | **Yes=1** | **No=0** | **Don’t remember=99** |  |
| *Hot dish №2* | Thai-style meat | **Yes=1** | **No=0** | **Don’t remember=99** |  |
| *Fruit platter* | Orange | **Yes=1** | **No=0** | **Don’t remember=99** |  |
|  | Sweet cherry | **Yes=1** | **No=0** | **Don’t remember=99** |  |
|  | Kiwi | **Yes=1** | **No=0** | **Don’t remember=99** |  |
|  | Green apple | **Yes=1** | **No=0** | **Don’t remember=99** |  |
|  | Red apple | **Yes=1** | **No=0** | **Don’t remember=99** |  |
| *Bread basket* | Fried bread rolls | **Yes=1** | **No=0** | **Don’t remember=99** |  |
|  | Rye bread | **Yes=1** | **No=0** | **Don’t remember=99** |  |
|  | Wheat bread | **Yes=1** | **No=0** | **Don’t remember=99** |  |
| *Desserts* | Honey cake | **Yes=1** | **No=0** | **Don’t remember=99** |  |
|  | Whoopee Pie cake | **Yes=1** | **No=0** | **Don’t remember=99** |  |
|  | «Molochnaya devochka» cake | **Yes=1** | **No=0** | **Don’t remember=99** |  |
|  | Cookies | **Yes=1** | **No=0** | **Don’t remember=99** |  |
|  | Candy | **Yes=1** | **No=0** | **Don’t remember=99** |  |
| *Beverages* | Zerno vodka | **Yes=1** | **No=0** | **Don’t remember=99** |  |
|  | Berkutti cognac | **Yes=1** | **No=0** | **Don’t remember=99** |  |
|  | Red wine | **Yes=1** | **No=0** | **Don’t remember=99** |  |
|  | White wine | **Yes=1** | **No=0** | **Don’t remember=99** |  |
| F. Clinical characteristics | | | | | |

| F1 | Did you feel sick after the event? | **Yes=1 No=0**  If no, stop the questionnaire |  |
| --- | --- | --- | --- |
| F2 | Date of symptom onset | ⬜⬜.⬜⬜.⬜⬜⬜⬜ ⬜⬜.⬜⬜  Day Month Year Time | |
| F3 | Did you seek medical attention | **Yes=1 No=0** |  |
| F4 | Were you hospitalized | **Yes=1 No=0** |  |
| F5 | If yes, date hospitalized | ⬜⬜.⬜⬜.⬜⬜⬜⬜  Day Month Year | |
| F6 | Which signs or symptoms did you experience | | |

| F7 | Fever | | | **Yes=1** | **No=0** | | | **Don’t remember=99** | |  | |
| --- | --- | --- | --- | --- | --- | --- | --- | --- | --- | --- | --- |
| F8 | If yes, maximum fever | | | ⬜⬜ ,⬜⬜ °C | | | | | | | |
| F9 | Stomachache | | | **Yes=1** | **No=0** | | | **Don’t remember=99** | |  | |
| F10 | Nausea | | | **Yes=1** | **No=0** | | | **Don’t remember=99** | |  | |
|  |  |  |  | **Yes=1** | **No=0** | | | **Don’t remember=99** | |  | |
| F11 | Fatigue | | | **Yes=1** | **No=0** | | | **Don’t remember=99** | |  | |
| F12 | Headache | | | **Yes=1** | **No=0** | | | **Don’t remember=99** | |  | |
| F13 | Chills | | | **Yes=1** | **No=0** | | | **Don’t remember=99** | |  | |
| F14 | Vomiting | | | **Yes=1** | **No=0** | | | **Don’t remember=99** | |  | |
| F15 | If yes: How many times a day | | | ⬜⬜ times a day | | | | | | | |
| F16 | Duration in days | | | ⬜⬜days | | | | | | | |
| F17 | Diarrhea | | | **Yes=1** | **No=0** | | | **Don’t remember=99** | |  | |
| F18 | If yes: How many times a day | | | ⬜⬜ times a day | | | | | | | |
| F19 | Duration in days | | | ⬜⬜days | | | | | | | |
| F20 | Other symptoms_________________________________________________ | | | | | | | | | | |
| F21 | Were you in contact with people with diarrhea within 7 days before you got sick? | | **Yes=1** | | | **No=0** | | | **Don’t remember=99** | |  |
| F22 | Do you know anyone who also got sick*?*  If yes, complete the table below | | **Yes=1** | | | **No=0** | | | If no, go to question F23 | |  |
|  | Full name | | **Degree of kinship** | | | **Address** | | | **Phone** | | Sick=1  No=0 |
|  |  | |  | | |  | | |  | |  |
|  |  | |  | | |  | | |  | |  |
|  |  | |  | | |  | | |  | |  |
|  |  | |  | | |  | | |  | |  |
|  |  | |  | | |  | | |  | |  |
| F23 | Were your family members present at the event? If yes, complete the table below | | **Yes=1** | | | **No=0** | | | If no, go to question F25 | |  |
|  | Full name | | **Degree of kinship** | | | **Address** | | | **Phone** | | Sick =1  No=0 |
|  |  | |  | | |  | | |  | |  |
|  |  | |  | | |  | | |  | |  |
|  |  | |  | | |  | | |  | |  |
|  |  | |  | | |  | | |  | |  |
|  |  | |  | | |  | | |  | |  |
| F25 | Have you been to any other event the week before you got sick/the wedding? (e.g., rehearsal dinner, bride’s farewell ceremony etc.) | | **Yes=1** | | | **No=0** | | | **Don’t remember=99** | |  |
| F26 | If yes, which events? *If no, finish the questionnaire* | |  | | |  | | |  | |  |
| F27 | 1 event___________________location______________when?__________ | | | | | | | | | | |
| F28 | 2 event_________________location_____________when?___________ | | | | | | | | | | |
| F29 | 3 event_________________location_____________when?___________ | | | | | | | | | | |
| F30 | *What other foods have you consumed?* | | | | | | | | | | |
|  | *Product groups* | *Response options* | | | | | | | | *Answer* | |
|  | Meat products | **Yes=1** | | | | | **No=0** | **Don’t remember=99** | |  | |
|  | Seafood | **Yes=1** | | | **No=0** | | | **Don’t remember=99** | |  | |
|  | Dairy products | **Yes=1** | | | **No=0** | | | **Don’t remember=99** | |  | |
|  | Eggs | **Yes=1** | | | **No=0** | | | **Don’t remember=99** | |  | |
|  | Chicken | **Yes=1** | | | **No=0** | | | **Don’t remember=99** | |  | |
|  | Duck | **Yes=1** | | | **No=0** | | | **Don’t remember=99** | |  | |
|  | Candy | **Yes=1** | | | **No=0** | | | **Don’t remember=99** | |  | |
|  | Confectionery products | **Yes=1** | | | **No=0** | | | **Don’t remember=99** | |  | |
|  | Salads | **Yes=1** | | | **No=0** | | | **Don’t remember=99** | |  | |
|  | Vegetables | **Yes=1** | | | **No=0** | | | **Don’t remember=99** | |  | |
|  | Baked goods | **Yes=1** | | | **No=0** | | | **Don’t remember=99** | |  | |

**Thank you for participation!**
