# Supplementary material for: Salmonellosis outbreak associated with the consumption of food at a wedding in an urban restaurant in Kazakhstan: a retrospective cohort study
Source: BMC Infect Dis. 2024 Dec 25;24:1464. doi: 10.1186/s12879-024-10382-4 (PMC11670359; doi:10.1186/s12879-024-10382-4)
Supplement: Supplementary file 2 — Supplementary Material 2 [file 12879_2024_10382_MOESM2_ESM.docx]

**Supplement 2.**

**Results of food and restaurant surfaces tested of an intestinal infection in an urban restaurant, Kazakhstan June 2022 (N=100)**

| **Sample (no. collected)** | ***E. coli*** | ***Enterobacter*** | ***S.* Enteritidis** |
| --- | --- | --- | --- |
| Food item | | | |
| Liver pancake | + | - | - |
| Mushrooms | + | + | - |
| Honey cake | + | + | + |
| Horsemeat from beshbarmak | + | + | - |
| Restaurant surface | | | |
| Preparation table | + | - | - |
| Serving table | + | - | - |
| Dining table | + | - | - |
| Dish rack | + | - | - |
| Plates from first course | + | - | - |
| Plates from second course | + | - | - |

*S.* Enteritidis, *Salmonella enterica* serovar Enteritidis
